# When demand exceeds supply: Liver transplantation due to alcohol use disorder in Austria

**DOI:** 10.1007/s40211-020-00364-8

**Published:** 2020-11-03

**Authors:** Stephan Listabarth, Andrea Gmeiner, Nathalie Pruckner, Sandra Vyssoki, Andreas Wippel, Daniel König

**Affiliations:** 1 Clinical Division of Social Psychiatry, Department of Psychiatry and Psychotherapy, Medical University of Vienna, Währinger Gürtel 18–20, 1090 Vienna, Austria; 2 Department of Health Sciences, St. Pölten University of Applied Sciences, Sankt Pölten, Austria

**Keywords:** Alcoholism, Liver cirrhosis, Epidemiology, Liver transplantation, Addiction medicine, Alkoholabhängigkeit, Leberzirrhose, Epidemiologie, Lebertransplantation, Suchtmedizin

## Abstract

**Background:**

Alcohol use disorder (AUD) is associated with a high prevalence rate and causes a significant burden on health systems globally. The most severe condition associated with AUD is end-stage alcohol-related liver disease (ARLD), for which liver transplantation (LTX) is the only curative therapy. However, the determination of key epidemiologic figures of both conditions is limited by several difficulties and challenges. Therefore, the goal of this paper is to discuss different epidemiological models to estimate AUD and ARLD prevalence, and compare the results of these models with LTX data.

**Methods:**

A literature search for epidemiological models estimating the prevalence of AUD and associated secondary diseases was conducted. Identified approaches are discussed and recalculated, applying the newest available data for Austria. The thus estimated numbers were, in a further step, set in relation to the national LTX statistics.

**Results:**

Besides health survey-based estimations and models based on economic data, estimations based on the mortality of ARLD (Jellinek formula) were identified. Depending on the prediction scenario, the calculated rates of prevalence of AUD ranged between 4.1% and 10.1% for the population aged older than 15 years. Furthermore, while the prevalence of secondary diseases due to AUD is high, only a marginal proportion (about 4%) of end-stage ARLD patients receive a new organ.

**Conclusion:**

These results suggest that the prevalence of AUD and associated diseases remain underestimated. Furthermore, a pronounced discrepancy between the number of ARLD deaths and the number of LTXs due to ARLD, and distinct regional differences in the supply of LTXs, were found.

## Abstract (de)

**Hintergrund:**

Eine Alkoholkonsumstörung ist eine der häufigsten psychiatrischen Erkrankungen und stellt global eine relevante Belastung der Gesundheitssysteme dar. Die schwerwiegendste Folgeerkrankung ist die Leberzirrhose, deren einzige kurative Therapie in einer Lebertransplantation (LTX) besteht. Die Ermittlung der epidemiologischen Kennzahlen für beide Krankheitsbilder ist jedoch durch etliche problematische Herausforderungen eingeschränkt. Ziel des Beitrags ist es daher, verschiedene epidemiologische Modelle, welche Prävalenz und Mortalität von Alkoholabhängigkeit und alkoholbedingter Lebererkrankung abschätzen, zu diskutieren und mit LTX-Daten zu vergleichen.

**Methoden:**

Eine Literaturrecherche für epidemiologische Modelle zur Abschätzung von Prävalenz der Alkoholkonsumstörung und der mit ihr verbundenen sekundären Morbidität wurde durchgeführt. Die identifizierten Ansätze werden diskutiert und unter Berücksichtigung der rezentesten Daten neu berechnet. Die so geschätzten Zahlen wurden in einem weiteren Schritt in Relation zu den nationalen LTX-Statistiken gesetzt.

**Ergebnisse:**

Neben auf Umfrage- und Wirtschaftsdaten basierenden Modellen wurden auf Daten zur Mortalität bei alkoholbedingten Lebererkrankungen beruhende Schätzungen identifiziert (Jellinek-Formel). Je nach Prognoseszenario lagen die berechneten Prävalenzraten für Alkoholabhängigkeit zwischen 4,1 und 10,1% für die Bevölkerung im Alter von über 15 Jahren. Zudem ist zwar die Prävalenz der alkoholkrankheitsbedingten sekundären Morbidität hoch, doch mit etwa 4% erhält ein nur ein ganz geringer Anteil der Leberzirrhosepatienten ein neues Organ.

**Zusammenfassung:**

Unsere Ergebnisse weisen darauf hin, dass die Prävalenz sowohl der Alkoholabhängigkeit als auch der damit assoziierten Morbidität nach wie vor unterschätzt wird. Weiterhin bestehen in Österreich hinsichtlich der alkoholbedingten Lebererkrankungen eine erhebliche Diskrepanz zwischen der Mortalitätsrate einerseits und der LTX-Rate andererseits sowie große regionale Unterschiede in der LTX-Versorgung.

## Introduction

With an estimated global prevalence rate of 1405 per 100,000 individuals, alcohol use disorder (AUD) represents the most common substance use disorder and places a significant burden on health care worldwide. Globally, harmful alcohol consumption and its associated comorbidities account for more than 3 million deaths (5.3% of all deaths) per year [1] and over 5% of all disability-adjusted life years (DALYs) [2]. Analyzing the current situation in Austria, data show that with an estimated prevalence of 1795 per 100,000 population, the burden of this disease exceeds the global average (1405 per 100,000) [1, 2]. The World Health Organization (WHO) reported 48% of all alcohol-related deaths to be attributable to liver cirrhosis in 2016, making it the most common cause of death among more than 200 known diseases associated with alcohol consumption. Besides its frequency within the population of patients suffering from AUD, liver cirrhosis as the final stage of alcohol-related liver disease (ARLD) is also of particular importance [3–5] as the already high frequency of ARLD among all liver diseases has been reported to be increasing for Europe and Central Asia [3]. Currently, the only curative treatment option available for decompensated liver cirrhosis is liver transplant (LTX) [3–5]. In Europe, about 7300 LTX were performed in 2017, 50% of which were necessitated due to cases of decompensated liver cirrhosis—up to 19% (*n* = 693) of those 50% attributable to AUD [6].

However, the actual determination of key epidemiologic figures, such as prevalence and mortality, is limited by several challenges: AUD is still associated with a high level of stigma within many societies [7–10]. This uncertainty not only impedes the evaluation in prevalence studies but also in clinical screening situations. Since, in contrast to diseases with distinct and objectively observable symptoms, the diagnostic criteria for AUD are almost exclusively based on self-disclosed information on behavior [11] and stigma-associated denial or understatement of drinking behavior is likely to occur. Furthermore, stigma associated with AUD may also reduce individuals’ willingness to seek treatment or medical advice. These factors are likely to cause an underestimation of the prevalence when health care data are used to determine the prevalence of AUD [12] and thus confound not only epidemiologic data of AUD itself but also those of secondary and associated diseases of harmful alcohol use, such as alcohol-related liver diseases (ARLD). Therefore, available epidemiologic data regarding AUD need to be interpreted with caution.

This paper aims to discuss different epidemiologic models estimating the prevalence of AUD and ARLD for a specific country or region, using Austria as an example, to further increase our understanding of epidemiologic data and to fully understand the scope of this disorder as well as associated secondary diseases. In addition, strengths and limitations of each model will be considered, and calculated numbers will be compared to national liver transplantation (LTX) data, with the latter being the sole relevant treatment option in patients suffering from ALRD.

## Methods

A literature search for epidemiological models estimating the prevalence of AUD and associated secondary diseases was conducted in Embase, Scopus and MEDLINE. Identified approaches were first evaluated for their strengths and weaknesses, and their results recalculated applying the newest available data. Anonymized data regarding all LTXs within Austria were extracted from the “Transplant Jahresbericht 2018” [13]—including the number of conducted LTXs, waiting list metrics and the number of donated organs. In a further step, these metrics were set in relation to the need for LTXs due to alcohol-related liver cirrhosis.

## Results

One of the most widely used models for estimating the rate of alcohol-dependent individuals in a specific population is the Jellinek formula. Already established in the late 1940s, this model estimates the prevalence of AUD based on population data regarding liver cirrhosis [14]. The following parameters are included in the model: *P*, indicating the share of liver cirrhosis attributable to alcohol abuse. *D*, specifying the death rate due to liver cirrhosis (whether caused by alcohol abuse or not). *K*, the death rate due to alcohol induced liver cirrhosis within the population of alcohol-dependent individuals. *R*, the ratio of all alcohol-dependent individuals to those with alcohol associated diseases. Using this formula, these quantities approximate *A*, the prevalence of AUD within a specific population. The Jellinek formula

$$A=\frac{P*D}{K}*R$$

can be used to estimate the prevalence of AUD within the population aged ≥15 (A), where

*P:* *share of liver cirrhosis attributable to alcohol abuse;*
*D:* *death rate due to liver cirrhosis whether caused by alcohol abuse or not;*
*K:* *death rate of alcohol-dependent individuals deceased due to alcohol-induced liver cirrhosis;*
*R:* *ratio of all alcohol-dependent individuals to those with alcohol-associated diseases.*

Jellinek had assumed *K, R* and *P* to be constant over time, whereas *D* was assumed to be the changeable and determining variable within this model. When applying this model to the most recent numbers in Austria—1304 deaths due to liver cirrhosis for a total population of 7,509,129 individuals in 2017 [15]—the model estimates an alcohol-dependent rate of 4.87% of the total population aged over 15 years.

However, several aspects of this model have been criticized [14, 16, 17], leading to decreased use in recent literature:

First, the assumption of handling the variables *K, R* and *P* as constant across time and independent of the geographic regions observed is subject of ongoing discussion and there have been attempts to adjust those variables for effects of time and region. Importantly, these ‘constants’ (*K, R* and *P*) exhibit high rates of variance: For example, the value of *P*, the share of liver cirrhosis attributable to alcohol abuse, had initially been determined to equal 50% by Jellinek. In contrast, the percentage postulated in previous literature varies from 29% up to 58%—depending on region/country as well as time point [18, 19]. Furthermore, for the variable *K*, the ratio of alcohol-dependent individuals to alcohol-dependent individuals with medical complications, values ranges from 4 to 5.3 in the literature [16].

Second, with *K, R* and *P* being treated as constants, *D* (the general liver cirrhosis death rate) is the primary influencing dimension in this model. However, this parameter itself is of limited reliability. While using data from the official national cause of death register—as in the previous example—may seem reasonable, it may also represent a source of bias: In the Austrian cause of death register, only one immediate cause of death may be coded, but, as a multitude of acute and chronic medical conditions associated with alcohol abuse may be present, this may promote inaccuracies and lead to an underrepresentation of deaths due to liver cirrhosis in the national register. Instead, death may be attributed to other, secondary but acute manifestations of gastrointestinal complications (e.g., esophageal varices bleeding) or infections (e.g., spontaneous bacterial peritonitis). Furthermore, previous literature has suggested that (alcoholic) liver cirrhosis may remain undetected in many cases, as this chronic disease has the trait to be asymptomatic for a prolonged period of time [20].

In order to adjust for the above-mentioned inaccuracies of the utilized variables of the Jellinek formula, the authors aimed to recalculate the model using data from the Global Burden of Disease Study (GBD) [3] which estimated 1860 deaths due to liver cirrhosis for 2017. This recalculation would then equate to a rate of alcohol-dependents within the population of 6.94% of all individuals aged over 15 years in Austria. When also adjusting *P* and *K*, as discussed in the preceding paragraphs, we predict a prevalence rate ranging from 4.1 up to 10.1% (Table 1).

**Table 1:** Estimates of the prevalence of alcohol-dependent individuals within the Austrian population aged ≥15 years (A) by the Jellinek formula according to data provided from Statistik Austria as well as the Global Burden of Disease Study (GBD) and displays the change in outcome parameters when data from GBD is adjusted for previously published data on either/or/and R/P

Modell	**P** (% of liver cirrhosis cases attributable to alcohol abuse)	**D** (death rate due to liver cirrhosis)	**K** (death rate of alcohol-dependent individuals deceased due to alcohol-induced liver cirrhosis)	**R** (ratio of all alcohol-dependent individuals to those with alcohol-associated diseases)	**A** (prevalence of alcohol-dependent individuals within the Austrian population aged ≥15 years) (%)
Statistik Austria 2017	*49*	*0.000174*	0.0070	4.00	*4.872*
GBD 2017	*49*	*0.000248*	0.0070	4.00	*6.936*
GBD 2017 1 (adjusted R^1^)	*49*	*0.000248*	0.0070	*5.30*	*9.190*
GBD 2017 2 (adjusted R^1^ and P^2^)	*58*	*0.000248*	0.0070	*5.30*	*10.878*
GBD 2017 3 (adjusted P^3^)	*29*	*0.000248*	0.0070	4.00	*4.105*

R^1^: according to Cleary [14]; P^2^: according to Nilsson et al. [19]; P^3^: according to Gunnarsdottir et al. [18]

The prevalence of cirrhosis and other chronic liver diseases due to alcohol use in Austria is estimated to be as high as 122,054 affected individuals in 2017. Recent data on the clinical course of alcoholic liver cirrhosis [20, 21] report a 1-year mortality of 17% to 56%—depending on the clinical constitution of the patient at time of diagnosis. When applying this data on the abovementioned prevalence number, we yield a range of 20,749 to 68,350 deaths per year due to ARLD. However, within the official Austrian death registry, only 582 deaths due to ARLD were reported for 2018 [15], and the GBD study [2] estimated a number of only 877 deaths due to ARLD for 2017.

Independent of the exact rate of prevalence or mortality, LTX undisputedly remains the only curative approach for advanced liver cirrhosis. When evaluating the most recent data on LTXs (not limited to a specific etiology), 182 transplantations (including living-donor transplantation) were conducted in Austria in 2018 [13], associated with a steadily increasing rate of LTXs, starting in 2010 and continuing until 2018. This increase is in stark contrast to all other rates of organ transplantations in Austria, where data on the recent incidence correspond to the roughly constant rates of transplantations in recent years. By the end of the year 2018, 87 individuals were on the waiting list for a liver transplant (45 in Vienna, 23 in Graz and 18 in Innsbruck). Of all individuals actively listed for a LTX between 2013 and 2018 in Austria (*n* = 1314), 66% could be provided with a new organ, 9% of the individuals deceased while on the list and the remaining 25% were either still waiting for transplantation, or their status changed due to worsening or improvement of their health status or personal choice. With 1.7 months on average until transplantation, the time on the waiting list for a liver transplant is the shortest compared to the duration on the waiting lists of all other organ transplants. However, also the average time until passing for individuals actively listed is the shortest (1.6 months on average) in comparison to waiting lists for other organ transplantations.

## Discussion

The actual prevalence of AUD and its secondary liver diseases is challenging to assess, despite several profoundly different approaches being employed. One typical approach is to use data of health surveys [22]. The most recent surveys report a total prevalence of 5% for the Austrian population aged 15 years or older (7.5% for males and 2.5% for females). In total numbers, this adds up to 365,000 individuals in Austria suffering from AUD. This approach, however, is limited by (1) selection bias, potentially leading to an underrepresentation of alcohol abusing individuals and those with increased consumption patterns in so-called representative study collectives, (2) conscious underreporting, and—in rare cases—also overreporting, of alcohol consumption and (3) inaccuracies in retrospective assessments of alcohol consumption—not necessarily by intent, but also due to the seemingly trivial nature or perceived insignificance of alcohol consumption in our daily lives [23].

Another major limitation of this approach is that, although providing a good overview of the average alcohol consumption of a population, neither does this data allow to infer a pattern of alcohol consumption on an individual level nor the frequency of AUD. This limitation is also valid for other approaches: estimating the prevalence by evaluating economic data, such as production/sales/import and export data regarding alcoholic beverages. While for countries with regulatory standards stipulating the publication of these data, such data may be seen as reliable and, thus, may be used in an effort to compensate for the limitations of health-survey-based approaches, this may not be true for all countries equally. In conclusion, estimations based on factors available on an individual level seem to be the most suitable, even though these approaches exhibit limitations as well.

In this paper, the Jellinek formula, as the most used approach to compensate for lack of valid data, was recalculated by the authors yielding results for the prevalence of AUD (4.87%) in line with the official report of the Austrian National Public Health Institute (Gesundheit Österreich GmbH) [23, 24]. Furthermore, aiming to correct for previously discussed and criticized assumptions and inaccuracies Jelinek’s formula is based on, the authors adjusted the model according to different scenarios suggested in previous literature [14, 16, 17]. These modified models produced results ranging from 4.1 up to 10.1%, depending on data sources and epidemiologic assumptions (Table 1).

The authors are fully aware that, due to the inherent limitations of the estimation of AUD prevalence rates derived from liver cirrhosis mortality data, the absolute calculated numbers should be treated with caution. However, the advantages of this approach over alternative ones have previously been mentioned in this paper. Furthermore, the intention of this model is not to provide an exact rate of prevalence, but rather to infer an estimate of the extent of this disease and the epidemiologic trends over time. In the national report on alcohol, Uhl et al. asserted a decreasing trend for the prevalence rate of AUD—among other reasons, also based on models applying the Jellinek formula [23]. In light of the mentioned limitations, it seems reasonable to ascribe this decline rather to progress made in the treatment and/or prevention of ARLD, the main parameter of this model, than an actual decline of the prevalence of AUD. A further limitation of the estimation of AUD is the use of estimated epidemiologic data—in the present case—data from the GBD study. In such studies, mortality and disease burden are often related exclusively to a specific disease and may therefore be overestimated. While one specific condition (e.g., AUD) may be of relevance, it is but one factor among many others responsible for the observed health status within the observed population [25].

When reviewing the relevant data regarding LTXs in Austria, it should be noted that the number of yearly transplantations has steadily increased—from around 150 in 2016 to more than 180 in 2018 [13]. However, the share of liver transplantations due to alcoholic liver cirrhosis amounted to only 22.98% (37 out of 161) of all LTXs conducted in 2017. While this is higher than the reported ratio of 19% observed across Europe for the time period of 1968 to 2016 [6], this has to been interpreted in context to the number of deaths in Austria caused by alcoholic liver cirrhosis and other chronic liver diseases due to alcohol use in the same year ranging from 582 [15] to 877 [2].

Thus, using cautious and conservative estimations, data suggest that only a minority of 4% to 6% of the ARLD patients in need actually receive a new liver. Concurrently, our conservative estimations signify that the overwhelming majority (94–96%) of patients in need do not have access to this life-saving treatment. This massive gap between demand and supply appears even more alarming when considering a significant number of deaths due to alcoholic liver cirrhosis may be unreported [24]. In addition, there are distinct regional differences in the availability and access to LTXs, which is not surprising when considering that highly specialized surgical centers are essential for these procedures. These structural differences may also be the reason for a drastic discrepancy in the number of conducted LTXs per population per region: 11.4 per million population per year for Lower Austria and 11.6 for Vienna versus 25.7 and 30.1 in Salzburg and Tyrol, respectively [13]. However, this difference may also further aggravate the general problem of undersupply with the only available curative treatment for (alcohol-induced) liver cirrhosis in specific regions.

In contrast to the most other indications for LTX, selection criteria for patients suffering of ARLD eligible to receive a transplant are vehemently discussed [26–30]. This is also due to the still widespread but obsolete concept of AUD being a self-inflicted condition rather than a disease worthy of treatment [31]. However, due to the fact of the scarcity of organs, this treatment option is only available for a select group of patients. Careful consideration of ethical and public health aspects are essential within this process [32]. Unfortunately, the evidence for unequivocally selection criteria is rather limited. For example, the often proclaimed 6‑month sobriety interval pretransplantation, which is used very frequently across multiple transplant centers, is arbitrary and not based on clinical evidence [33]. Although there is a small minority relapsing among those receiving a new liver, it is doubted that alcohol consumption behavior and sobriety duration prior to LTX is in any way associated with the overall outcome, i.e. patients’ survival [31, 34]. Even for patients suffering from alcoholic hepatitis or acute-on-chronic liver failure, no decrease in survival rate was found when compared to a control group of patients transplanted due to any other indication than ARLD [35, 36]. A further aspect, in regard to organ allocation, is that the often-vague policies vary from country to country. Whereas in, for example, Germany, the comparatively detailed guidelines (“Richtlinie gemäß § 16 Abs. 1 S. 1 Nrn. 2 u. 5 TPG für die Wartelistenführung und Organvermittlung zur Lebertransplantation”) do not only provide specific criteria for allocation of donor organs (e.g., 6‑month abstinence rule for LTXs due to alcoholic liver cirrhosis) but also specify the role and constitution of boards for every transplant center and an additional nation-wide additional panel of experts for exceptional cases, the situation is less clear in other countries within the European region (e.g., Austria), where exact legislative guidelines regarding transplantation allocation or a transplantation board are often lacking and each transplantation center is obliged to define allocation criteria by itself (“Verfahrensanweisung nach §10 OTPG”).

## Conclusion

Besides health survey-based estimations and models based on economic data, the Jellinek formula, an estimation based on the mortality of ARLD, is an approach to determine the prevalence of AUD in specific populations. The yielded results for Austria suggest that AUD may still be an underestimated problem. Furthermore, the considerable gap between deaths due to ARLD and the number of conducted LTXs due to ARLD is alarming, as well as the distinct regional differences in the supply of LTXs in between Austrian states. The shortage of donor organs highlights the importance of early detection and intervention in AUD prior to the emergence of ARLD.
